# TimeREISE: Time Series Randomized Evolving Input Sample Explanation

**DOI:** 10.3390/s22114084

**Published:** 2022-05-27

**Authors:** Dominique Mercier, Andreas Dengel, Sheraz Ahmed

**Affiliations:** 1 German Research Center for Artificial Intelligence (DFKI), 67663 Kaiserslautern, Germany; andreas.dengel@dfki.de (A.D.); sheraz.ahmed@dfki.de (S.A.); 2 Department of Computer Science, TU Kaiserslautern, 67663 Kaiserslautern, Germany

**Keywords:** deep learning, time series, interpretability, explainability, attribution, convolutional neural network, artificial intelligence, classifications

## Abstract

Deep neural networks are one of the most successful classifiers across different domains. However, their use is limited in safety-critical areas due to their limitations concerning interpretability. The research field of explainable artificial intelligence addresses this problem. However, most interpretability methods align to the imaging modality by design. The paper introduces TimeREISE, a model agnostic attribution method that shows success in the context of time series classification. The method applies perturbations to the input and considers different attribution map characteristics such as the granularity and density of an attribution map. The approach demonstrates superior performance compared to existing methods concerning different well-established measurements. TimeREISE shows impressive results in the deletion and insertion test, Infidelity, and Sensitivity. Concerning the continuity of an explanation, it showed superior performance while preserving the correctness of the attribution map. Additional sanity checks prove the correctness of the approach and its dependency on the model parameters. TimeREISE scales well with an increasing number of channels and timesteps. TimeREISE applies to any time series classification network and does not rely on prior data knowledge. TimeREISE is suited for any usecase independent of dataset characteristics such as sequence length, channel number, and number of classes.

## 1. Introduction

The success of deep neural networks comes from the superior performance and scaling deep neural networks offer compared to traditional machine learning methods [1]. However, during the last few decades, the need for explainable decisions has become more significant. In critical infrastructures, it is inconceivable to use approaches without any justification for the results [2]. In the medical sector, financial domain, and other safety-critical areas, explainable computations are necessary by law [3]. Furthermore, there are ethical constraints that limit the use of artificial intelligence even more [4,5]. Accordingly, a large research domain evolved. This domain covers explainable artificial intelligence (XAI). One main goal is to propose techniques that provide interpretable results to enable the broader use of deep neural networks.

For several years researchers developed modifications of the networks and model agnostic methods to provide these results [6]. The majority of these methods originate from the imaging modality as its concepts are easier to interpret for humans [7]. Model agnostic methods have shown especially great success. One famous category of model agnostic approaches is attribution methods [8]. The number of available methods in this category increases every year. One advantage of them is their loose coupling with the network. In addition, they do not limit the processing capabilities of the network, although some attribution methods include minor limitations concerning the network architecture. The downside of these methods is that the provided results require additional human inspection and interpretation. Furthermore, they do not make any statement related to the concepts covered by the network. Revealing the concepts learned by the network is not the goal of these approaches. Considering the time series modality, this is not a huge drawback, as concepts are not well defined in this domain, and an explanation based on pre-defined concepts would not be suitable.

Despite their great success, not all of these methods can be applied to time series. Besides the above-mentioned limitations, additional properties arise in the time series context. These properties are less important for the imaging modality, but they are pivotal for the success of an attribution method in the time series context. Noisy explanations are acceptable in the image domain but can result in low information gain in time series interpretability. Another aspect is the Continuity of the attribution [9]. It is pivotal for time series attributions that a certain degree of Continuity is preserved. In the paper written by Crabbe and Van der Schaar [10], the aspect of Continuity is addressed as relevant for the interpretability of an attribution map as the Continuity can greatly lower the cognitive effort required to understand an attribution map. In addition, an explanation that includes large spikes within important data points within small windows introduces ambiguity and increases the cognitive load. Due to the possible infinite length and number of channels, it is unavoidable to focus on every data point. The explanation needs to highlight the significant time frames and channels. This is not the case in the image domain, as the number of channels and their roles are pre-defined. The channels in the image domain are used together, which is not possible in the time series domain.

Taking into account the above-mentioned limitations and time series specific properties there is no perfect attribution method available for time series. This paper proposes TimeREISE, an instance-based attribution method applicable to every classifier. It addresses common bottlenecks such as runtime, smoothness, and robustness against input perturbations as mentioned in [11]. Many methods suffer from large computation times making them unfeasible for real-time applications. Another aspect mentioned by Mercier et al. covers noise included in the explanation, making it difficult to interpret the maps. The rest of the paper shows that the explanations provided by TimeREISE are continuous, precise, and robust. Without prior knowledge about the dataset, it is possible to produce attribution methods with different granularity and smoothness. The idea origins from RISE [12] and various perturbation-based attribution methods. Two main advantages are the following: TimeREISE is applicable to backbox classifiers, and its runtime does not scale directly with the input shape of the data.

## 2. Related Work

Interpretability methods are widespread across the different modalities such as image, natural language, and time series. A good overview of the diversity of these methods is given by Das and Rad [8]. Independent of the modality, the goal is to identify a significant subset of features to overcome ethical and industrial restrictions as mentioned by Peres et al. [2] and Karliuk [4]. Furthermore, the identification of these features must be precise and easy to understand. Therefore, noise free explanations that pass a sanity check are required. One prominent class of interpretability methods are attribution techniques. The following paragraphs describe the different attribution methods, their categories and characteristics. These methods are used to compare them to TimeREISE and they cover a broad set of post-hoc attribution map approaches applicable to time series classification.

The first sub-category of attribution methods covers the gradient-based approaches. A good survey of these was provided by Anacona et al. [13]. These methods use backpropagation to compute the importance of the features. Speaking of the advantages and disadvantages of these methods, they are known for their superior runtime but suffer from noisy gradients and access to the model internals. Guided-Backpropagation and IntegratedGradients are two well-known gradient-based methods. Guided-Backpropagation computes the gradient concerning the target prediction based on the non-negative gradients. More information about this approach was provided by Sundararajan et al. [14]. IntegratedGradients uses so-called baselines and approximates the integral of the gradients compared to the baseline. Further information is given by Springerberg et al. [15].

In contrast to these methods, the perturbation-based techniques do not require full access to the model as they perturb the input. A disadvantage of these methods is the increase in time as they utilize multiple forward passes. One famous example is the FeatureAblation presented in Fisher et al. [16]. Therefore, the features get replaced with a baseline value such as the mean. Next, the prediction is used to evaluate the impact. Very similar to this approach is the Occlusion presented in Zeiler et al. [17]. The features are removed completely.

The last category covers methods that do not fit directly to the previously mentioned. One method that falls into this category is LIME, introduced by Ribeiro et al. [18]. Although LIME performs perturbations to the input it is different in a way that a local model is trained to estimate the importance.

To evaluate the effectiveness of attribution maps a set of well-known metrics evolved. An important fact is that the ground truth of the feature importance is not given in most cases, and the measurements have to deal with that. One approach is to perform a deletion and insertion test as both are two well-known techniques to evaluate the efficiency of attribution methods. Petsiuk et al. [12] used them to provide evidence for their attribution method. Another well-known approach is to use the Infidelity and Sensitivity proposed by Yeh et al. [19]. To compute the Infidelity, the attribution gets perturbed by a significant amount, and the prediction change is evaluated. The Sensitivity perturbs the input by an insignificant amount, and the attribution is compared to the original one. A third metric related to robustness is Continuity. A continuous attribution map may suffer in the insertion evaluation, however, smooth attribution maps are more robust against attacks. Detailed information about the adversarial robustness was given by Alvarez et al. [20]. In addition, smooth attribution maps require less cognitive effort for interpretation as stated by Abdul et al. [9]; however, the correctness of the method needs to be preserved [21]. It has been shown that existing methods such as Guided-Backpropagation and IntegradedGradients can act as edge detectors when the network is randomized, resulting in misleading explanations. Finally, one of the most important aspects is the scaling concerning the runtime, as this defines the usability.

## 3. TimeREISE

**Figure 1:**
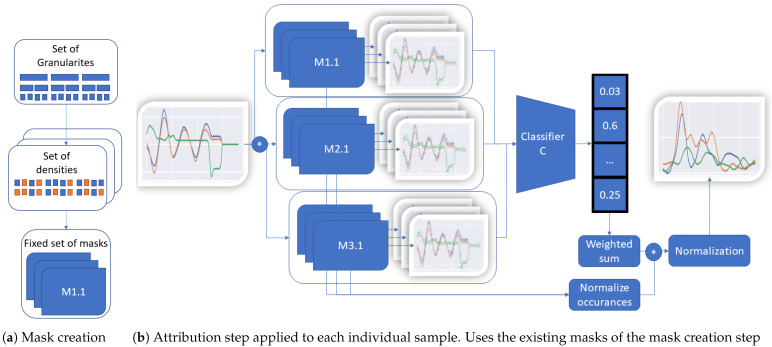
TimeREISE. Shows the two steps required to use TimeREISE. (**a**) shows the generation based on a set of different granularities and densities. The granulariy defines the number of slices and the density of the number of perturbed slices within a mask. (**b**) shows a set of masks (M1.1 to M3.x) applied to the input using an exchangeable perturbation function. The default perturbation is an element.wise multiplication. The masked input is passed to a classifier and the classification score is retrieved. The classification score is multiplied (*) by the masks and normalized by the number of feature occurrences. Finally, the attribution is normalized based on the number of occurrences of each point.

This paper presents the novel approach TimeREISE, a post-hoc interpretability method applicable to any classification network. The work was inspired by Petsiuk et al. [12]. They presented a random perturbation-based approach for the image domain used as a baseline to build TimeREISE. Similar to RISE [12] masks are generated, applied to the input, and the output confidence is measured using the classification scores. However, there are several adaptations in the native RISE [12] to enhance the approach and successfully apply it to time series data. Besides the simple normalization based on the occurrences of each data point, TimeREISE extends this to create masks that evaluate the different channels. Therefore, the masks cover the time and channel direction. This makes it possible to evaluate different combinations, e.g., the first timesteps are perturbed for the first channel and the second half of the timesteps are perturbed for the other channel. The second main addition applied is the summation over different probabilities. RISE [12] uses only a fixed probability of occluded points to create the masks resulting in a fixed density. In contrast to that, TimeREISE uses masks of different densities and combines them in an additive manner which removes the assumption of the number of relevant data points. Finally, the granularity is introduced as a parameter to define granularity of the explanation. This directly affects the size of the pattern analyzed. Figure 1 shows the overall workflow of TimeREISE. M denotes a set of masks, e.g., M1.x can have a smaller window size related to a finer granularity. M3.x can have a larger windows size for coarse patterns. This makes it possible to cover smaller and larger patterns within the data. The same holds for the density, e.g., M2.x could have the same granularity as M1.x but with a more dense or sparse feature perturbation. In Figure 1a the mask generation is shown. The process to create the masks can be done once for the dataset properties as it only depends on the number of timesteps, channels and the provided set of granularites and densities. The process shown in Figure 1b needs to be executed for each sample.

### 3.1. Mathematical Formulation

TimeREISE extends the native mathematical formulation presented by Petsiuk et al. [12] utilizing the different channels. TimeREISE generates masks with the shape $s^{\prime }=\left(c,t^{\prime }\right)$ instead of $s^{\prime \prime }=\left(1,t^{\prime }\right)$ where $t^{\prime }$ refers to the downsampled time axis and *c* to the channels. This enhances TimeREISE to apply masks that occlude different timesteps $t^{\prime }$ across all channels *c* within a mask $s^{\prime }$ instead of using the same timesteps $t^{\prime }$ across all channels *c* as it is the case for $s^{\prime \prime }$. Furthermore, the Monte Carlo sampling is performed across a set of densities *P* and granularities *G*. This enhances the masks to consider several density values *p* to regularize the density of the attribution. Similarly, the use of several granularity values *g* regularizes the size of the occluded patches. This changes the set of masks as shown in Equation (1).

(1) $$M=\{M_{0}^{p,g},\ldots ,M_{N}^{p,g}|p\in P\wedge g\in G\}$$

Finally, denote *S* as the weighted sum of the scores produced by the network and the random masks *M* similar to Petsiuk et al. but normalize each feature as shown in Equation (2).

(2) $$S=\sum_{c=0}^{C}\sum_{t=0}^{T}\frac{S_{c,t}}{\sum_{m=0}^{N}M_{c,t,n}}$$

### 3.2. Theoretical Correctness

Concerning sanity checks mentioned by Adebayo et al. [21], the correctness of the approach is crucial, and it should mainly depend on the learned behavior rather than highlighting dataset-specific features. Adebayo et al. showed that in the image domain the methods may produce edge detectors or show similar attribution maps when some weights of the networks are randomized. However, this mainly holds for gradient-based methods. TimeREISE does not suffer from this as it only depends on the logits produced by the network prediction. The complete process only depends on the forward pass of the network and has no access to any internal parameters. Therefore, the attribution only relies on the prediction of the network. This prediction depends on the correct weights of the neurons.

### 3.3. Theoretical Runtime

For the runtime evaluation, initialization and attribution two separate processes are considered. Equation (3) shows the runtime to create the set of masks for a given set of density probabilities *P*, granularities *G* and the number of masks *N* defined for each combination of $p_{i}$ and $g_{i}$. β is defined as the constant time to create the given map. In addition, *P* and *G* are independent of the data shape and therefore do not increase and can be considered as constant factors leading to a runtime of Θ(N).

(3) $$t_{init}=P*G*N*\beta \to t_{init}=\Theta \left(P*G*N\right)\to t_{init}=\Theta \left(N\right)$$

Equation (4) shows the linear runtime of the attribution step. γ is defined as the constant time to apply the perturbation and δ as the constant time the classifier requires to forward pass the sample. Similar to the initialization step, *P* and *G* are assumed as constants resulting in a runtime of Θ(N).

(4) $$t_{apply}=P*G*N*\gamma *\delta \to t_{apply}=\Theta \left(N\right)$$

In the following section, the runtimes of existing methods are explained. As the runtime can heavily depend on the implementation and the used hardware, a theoretical analysis offers a much more accurate analysis. The gradient-based methods such as GuidedBackprop are superior concerning their runtime. These methods only depend on the backward pass, which is executed once for the GuidedBackProp [15] and n_step times for the IntegratedGradients [14]. This means that both do not depend on the number of channels or timestep if we assume that the time for a backward pass is constant. Perturbation-based methods usually require multiple forward passes. Their runtime depends on the parameters that affect the number of forward passes. For Occlusion [17] and FeatureAblation [17] this is the window size. Using the same window size for a longer sequence results in more forward passes. Depending on the window size, the number of passes can be adjusted. Finally, the runtime of LIME [18] depends on the number of samples used to train the surrogate model to estimate the importance of the different features for the given samples. This means that similar to the perturbation-based approaches it depends on the channels and timesteps. To get a precise model a larger feature space requires more samples to be processed by LIME.

As described above, the runtime of TIMEREISE mainly depends on the number of masks used to compute the attribution map. This number can vary based on the time series, granularity and density. Using hyperparameter tuning it is possible to find the minimal number of required masks to produce a map that shows only insignificant changes.

### 3.4. Theoretical Implementation

The implementation of TimeREISE can be divided into two parts similar to the RISE [12] implementation by Petsiuk et al. [12]. In the first stage shown in Algorithm 1, a set of masks suited for the input shape gets generated. This has to be executed only once per dataset. Therefore, consider every combination of probabilities *P* and granularities *G* provided. The probability refers to a threshold used to determine the density of the mask. Granularity refers to the amount of data considered in a single slice. The downsampling and upsampling are performed along the time axis. Uniform refers to a uniform distribution with the given shape $s^{\prime }$. An additional cropping step is performed to preserve the original shape *s*.

Algorithm 2 performs the actual attribution. A predefined perturbation method σ is applied to the input *x* using every mask $m_{i}$ and is passed to the classifier θ. As default perturbation, the method uses the simple element-wise multiplication of the input *x* and the mask $m_{i}$ as proposed by Petsiuk et al. This results in a list of scores stored in *S*. Next, the matrix product of $S_{T}$ and the masks *M* is computed and each point is normalized by the number of occurrences *N* in the set *M*. Finally, the map is normalized to values between zero and one.
**Algorithm 1** Mask generation-Initialization.1:Define: *s* as input shape, *P* as set of probabilities, *G* as set of granularities for timesteps, *N* number of masks and *M* as list of masks.2:**for**p=1,…,P  **do**3:     **for** g=1,…,G  **do**4:         **for** i=1,…,N  **do**5:           $s^{\prime }=downsample\left(s,g\right)$6:           $m=uniform(s^{\prime })<p$7:           m=upsamle(m,s)8:           m=crop(m,s)9:           Append *m* to *M*10:         **end for**11:     **end for**12:**end for**13:$S=\frac{S\times M}{N}$14:$S=\frac{S-\min(S)}{\max(S-\min(S))}$

**Algorithm 2** Mask application-Attribution.
1:Define: *x* as input, θ as classifier, σ as perturbation function, *S* as list of scores and *N* as feature occurrences across all masks *M*.2:
**for** m=1,…,M **do**
3:    $x_{m_{i}}=\sigma \left(x,m_{i}\right)$4:    $y^{\prime }=\theta \left(x_{m_{i}}\right)$5:    Append $y^{\prime }$ to *S*6:
**end for**
7:

$S=\frac{S^{T}\times M}{N}$

8:

$S=\frac{S-\min(S)}{\max(S-\min(S))}$




## 4. Datasets

The work uses multiple datasets from the well-known UEA & UCR repository [22] to perform the experiments. The selection of datasets is based on a sufficient number of samples and the dataset modalities such as the number of timesteps, channels, and classes. Very small datasets with less then 300 samples were excluded as this would result in difficulties when training the network and lead to more variance concerning the metrics. Furthermore, the list of datasets is extended using the Anomaly dataset proposed by Siddiqui et al. [23]. This synthetic dataset serves as an interpretable baseline as the point anomalies in this dataset are mathematically defined, and therefore the ground truth attribution is available. Conversely, this is not the case for the other datasets, and only limited interpretability is given. Table 1 lists the datasets and their characteristics. These are assigned to the critical infrastructure domains they belong.

## 5. Experiments

The following paragraph describes the general setup to reproduce the results and covers decisions that affect the experiments. Following the generic experiment setup, the paper provides experiments on the insertion and deletion of data based on the importance scores of the attribution methods, an Infidelity and sensitivity analysis, and visual examples of the method and other state-of-the-art attribution methods. In addition, the experiments cover a sanity check to validate the correctness of the approach and runtime analysis to evaluate the dependency on the dataset properties such as channels and timesteps.

### 5.1. Baseline Accuracy

**Figure 2:**
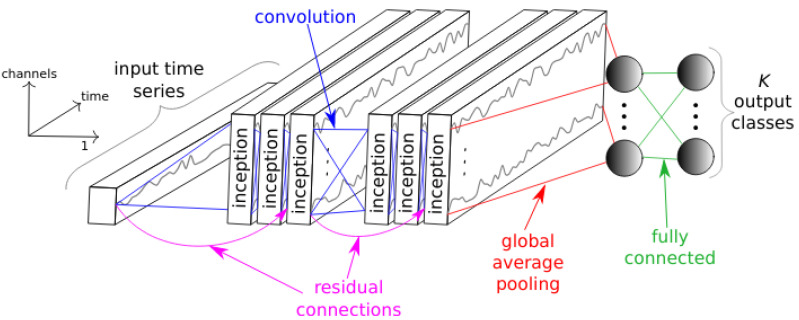
InceptionTime. Shows the general architecture of InceptionTime proposed by Fawaz et al. [24]. The architecture shows that there are several inceptionblocks consisting of convoluition layers. Furthermore, the network has residual connections to skip some inceptionblocks. After the last inceptionblock there is a global average pooling followed by a fully connected layer to produce the output classification. Figure taken from [24].

As model InceptionTime the current state-of-the-art proposed by Fawaz et al. [24] was used. The network architecture consists of multiple inception blocks followed by a global average pooling and a fully connected layer. Each inception block consists of multiple convolutional layers and a max-pooling. Furthermore, the network uses residual connections between the input and the different inception blocks. It is based on the Inception-v4 [25] architecture but specifically aligned to the time series domain and has shown to achieve good performances across time series classification datasets while being very robust. Figure 2 shows the architecture of IncpetionTime. For detailed information about the InceptionTime, the reader is referred to the paper of Fawaz et al. [24].

The network was trained using a learning rate scheduler to half the learning rate on plateaus and early stopping to prevent overfitting. As an optimizer, SGD was applied with an initial learning rate of 0.01 and a maximum of 100 epochs. None of the datasets required more than the 100 epochs, as the experiments have shown that every model converged in less than 100 epochs. As some datasets are very large, and the computation of measures such as the Sensitivity is computationally expensive, this work randomly sampled a set of 100 test samples to perform the attribution on a representative subset. In addition, the base accuracy scores for the whole datasets and the subset are provided in Table 2 which highlight that the findings, based on the subset, can be transferred to the complete datasets. Concerning the attribution methods GuidedBackprop [15], IntegratedGradients [14], FeatureAblation [17], Occlusion [17] and LIME [18] were used as state-of-the-art methods. This set of methods covers all categories of attribution methods mentioned in Section 2.

### 5.2. Sanity Check

**Figure 3:**
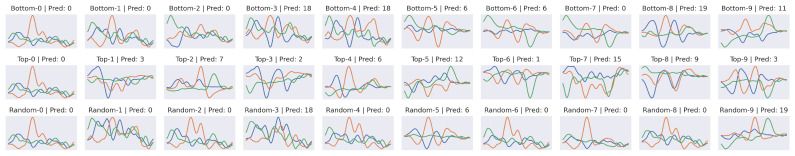
Sanity Attribution. Shows the attribution map of a CharacterTrajectories sample. Top-X: top-down randomization of X layers, Bottom-X: bottom-up randomization of X layers. Random-X: randomized layer/block X. Top-0 refers to the original attribution map. The results provide evidence that the TimeREISE method strongly depends on the model parameters.

**Figure 4:**
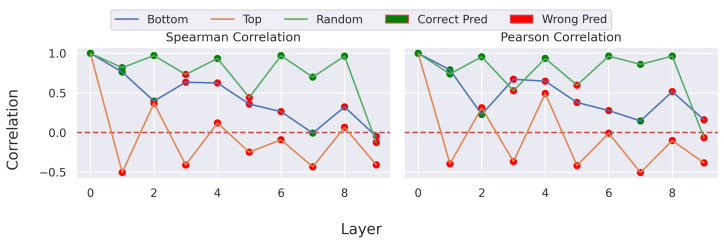
Sanity Correlation. Shows the correlation between the maps with randomized layers and the original attribution map. Refers to the attribution maps shown in Figure 3. The figure shows that changes to the network result in a lower correlation of the attribution map compared to the original attribution map.

In addition to the theoretical explanation of the correctness, a sanity check was conducted. Therefore, a sample of the CharacterTrajectories dataset was used, and the attribution map for different states of the model was computed. In Figure 3 the different attribution maps are shown. The first column always shows the original attribution map. Going from left to right increases the number of randomized layers for the top-down and bottom-up approaches. The first row refers to the bottom-up approach in which the layers are sequentially randomized starting from the first convolutional block in the first inception block up to the last dense layer. Respectively, the second row shows the top-down approach randomizing the dense layer first and the first convolutional block of the first inception block last. The third row covers the independent randomization of a single layer in the case of ‘Random-9’ as it refers to the last dens layer and single block for the other cases. Across all setups, it is visible that randomizing the layer weights results in significant changes in the attribution map. In Figure 4 the spearman and Pearson correlation between the original and randomized attribution map is given. The Spearman correlation was used as it is a rank-based approach and provides information about the preservation of the ranks of the points within the sample. The color of the individual points shows the correctness of the prediction using the manipulated network. It is visible that the correlation of the attribution maps with the correct prediction is higher than for others. This shows that TimeREISE successfully depends on the prediction of the network. Furthermore, the figure shows that for the top-down randomization the correlation drops by a large value. Similarly, the bottom-up randomization shows an increasing drop in correlation. However, randomizing a single layer or block resulted in higher correlation values except for the randomization of the last dense layer. This can be explained by the structure of InceptionTime, as it is very robust concerning the randomization of a single block. This is further validated by the correct predictions within this setup.

### 5.3. Runtime Analysis

**Figure 5:**
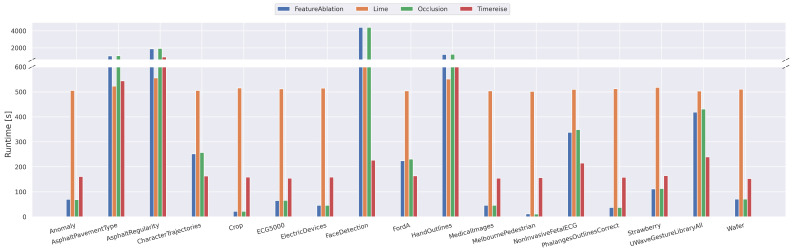
Runtime Real Data. Shows the runtime on the real datasets used in all experiments below. The runtime is given in seconds for 100 attribution maps. TimeREISE shows a stable runtime across all datasets. For long-sequence datasets the runtime of the other approaches increases.

**Figure 6:**
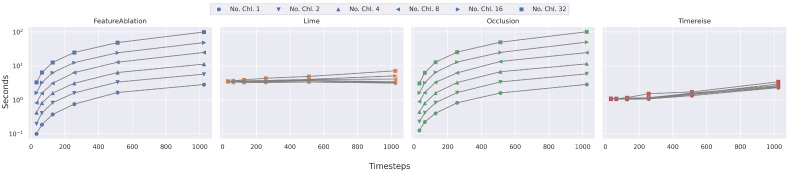
Runtime Parameter dependency. Shows the runtime on synthetic data with different number of timesteps and channels. IntegratedGradients and GuideBackprop are excluded. The runtime of FeatureAblation and Occlusion increase dramatically with the longer sequences. TimeREISE and Lime show a stable runtime.

Figure 5 shows the runtimes for the experiments executed on 100 samples. The results show that methods that directly depend on a window size, such as FeatureAblation [17] and Occlusion [17], require much longer processing for the datasets that have a large number of features. Especially, the runtimes for the four datasets with the highest number of channels and timesteps highlight that the increasing feature number makes them unsuitable for some cases. To reduce the processing time it is possible to select a larger window, however this requires knowledge about the dataset and the size of the pattern within the dataset. In contrast to that, GuidedBackprop [15] and IntegratedGradients [14] are excluded as they do not depend on such properties of the datasets. LIME [18] and TimeREISE mainly depend on the number of samples and masks defined for each of the approaches. The experiments show that in both cases, the runtime is constant for the parameters that were selected. However, as mentioned it would be possible to finetune the parameters. In addition to the real datasets, Figure 6 shows the increase based on the number of timesteps and channels. These results refine the previous analysis. The FeratureAblation and Occlusion increase based on the timesteps and the channels, whereas the processing time of LIME and TimeREISE only slightly increase as the forward passes require more time. This holds for the gradient-based methods and the backpropagation too. The difference is that the number of backward passes required in those methods is limited, whereas the number of forward passes for LIME and TimeREISE is higher. It has to be mentioned that the number might vary depending on the implementation and hardware, however they provide insights into the expected behavior when changing the dataset.

### 5.4. Insertion & Deletion

The causal metric was used by Fong and Vedaldi [26] to explain the significant values of an attribution method. The intuition behind the deletion is that the prediction of a classifier changes if the cause gets removed. This applies to the insertion as well. In the case of the deletion, the points starting with the most important one are removed from the input, and the prediction gets computed. Large drops suggest that the feature was significant for the prediction. Further, the AUC based on the sequential deletion of features to rank the methods across every dataset was computed. In the case of the deletion, lower AUCs suggest that the method is superior in spotting important parts of the input. Similarly, the same was done for the insertion, starting with a sample that has only mean values. For the insertion, higher AUCs are superior. Large increases in this setup correspond to adding important data points relevant to the prediction.

**Figure 7:**
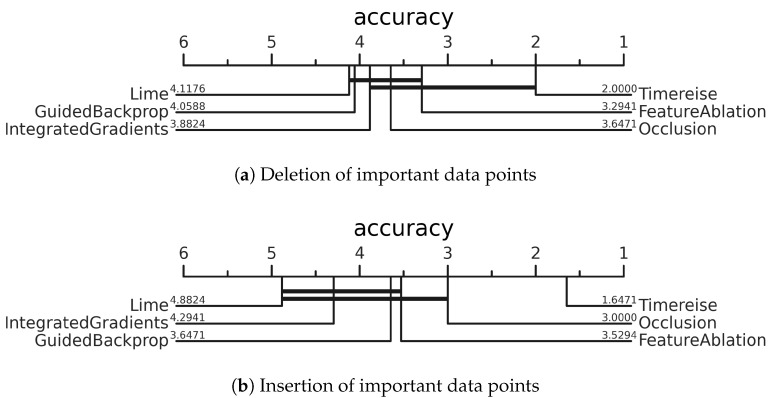
Deletion & Insertion. Critical difference diagram showing the average rank of each attribution method across all datasets. The ranking is based on the AUC using accuracy. Perturbation-based approaches achieve better results on the deletion and insertion test. TimeREISE shows a superior performance for both tests.

Figure 7 shows the critical difference diagrams of every attribution method. These were calculated using the AUC based on the achieved accuracy. In Figure 7a TimeREISE shows an outstanding performance compared to the other state-of-the-art methods with respect to the deletion of significant data that affects the classifier performance. Another important finding is that the methods that utilize a window, such as FeatureAblation and Occlusion show better performances concerning the deletion compared to methods that directly depend on the gradients such as GuidedBackprop and IntegratedGradients. However, Figure 7b highlights that the results are the same for the insertion task. One reason for its outcome is the smoothing applied to approaches that use a defined window. Gradient-based methods provide noisy and spiking attribution maps.

Table 3 shows the different results of the deletion and insertion for every individual dataset. Furthermore, the table provides the average scores achieved by the methods. TimeREISE shows a superior behavior in both the average deletion and insertion score. The method achieves the best (lowest) score for 13 datasets and an average of 0.2516. The second-best approach concerning the average AUC score is GuidedBackprop with a score of 0.3220 and two times the best performance. While TimeREISE has the best average score for the insertion, it scores only two times the performance. GuidedBackprop achieves five times, IntegratedGradients four times and Lime three times the best score in the insertion task. However, the average score of TimeREISE is 0.7510 compared to the second-best of 0.6653 for the Occlusion.

### 5.5. Infidelity & Sensitivity

The Infidelity and Sensitivity proposed by Yeh et al. [19] cover significant and insignificant changes applied to the attribution and the input. The intuition behind Infidelity is that a significant perturbation of the attribution map leads to a change in the prediction. Similarly, the Sensitivity is calculated using an insignificant change in the input sample. The Sensitivity requires to recompute the attribution maps. For both Infidelity and Sensitivity, lower values are better. For Infidelity, 1000 perturbations were computed for each of the 100 samples and computed the averaged Infidelity value. In addition, 10 perturbations for each of the samples were computed, and their Sensitivity was calculated.

Starting with Infidelity, the results shown in Table 4 emphasize that there is no significant difference between the different methods. Overall the average scores differ only by 0.011, which is an insignificant difference. Across all datasets, the methods perform similarly, and it is impossible to create a critical difference diagram as the null hypothesis does hold. Interestingly, the Infidelity scores for the ElectricDevices and PhalangesOutlinesCorrect dataset are much larger compared to those of any other dataset.

The Sensitivity experiments are shown in Table 5. The results of these experiments show a significant difference between the methods. The best result was achieved by TimeREISE, with a score of 0.0533. The worst result was achieved by LIME, with a score of 0.2182, which is about four times larger than the score of TimeREISE. The overall finding was that the perturbation-based approaches are superior in the case of Sensitivity compared to the gradient-based or others. This is the case as the gradient-based methods result in noisy attribution maps, whereas the perturbation-based come up with smoothed maps based on a window of multiple features. This smoothing increases the robustness against minor changes in the input.

**Figure 8:**
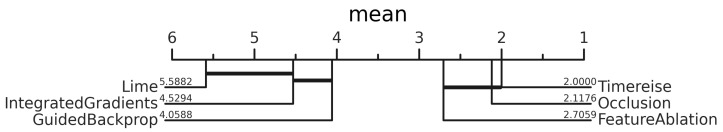
**Sensitivity.** A critical difference diagram showing the average rank of each attribution method across all datasets. The ranking is based on the average Sensitivity. Perturbation-based approaches show superior performance. The horizontal bars show the groups that can be concluded based on the rank of the methods. They clearly highlight the high and low performing sets.

In Figure 8 the critical difference diagram across all datasets is provided. It shows the superior performance of the perturbation-based approaches compared to the other approaches. In addition, it highlights that TimeREISE is only slightly above the Occlusion method.

### 5.6. Attribution Continuity

Furthermore, this work calculated the Continuity proposed by Abdul et al. [9]. Continuity is a measurement that bridges correctness and visual interpretability. The Continuity for each feature was calculated as presented in Equation (5) and took the mean for the overall evaluation between the methods. Lower values are better with respect to the cognitive load but might conflict with the exact correctness of the feature importance.

(5) $$C=\sum_{c=0}^{C}\sum_{t=0}^{T-1}|S_{c,t}-S_{c,t+1}|$$

In Table 6 we show the average Continuity of the attribution methods. Similar to the Sensitivity, smaller values are better. Interestingly, the performance of the attribution methods is very similar to the Sensitivity. Again TimeREISE shows superior performance with a score of 0.0267 compared to Occlusion as the second-best approach with a score of 0.0565. The reason for the superior performance is the smooth mask design. The masks of TimeREISE are created on a downscaled sample, and then they are upscaled using interpolation to the original input size. This results in smoother masks compared to Occlusion and FeatureAblation, which utilize fixed windows.

**Figure 9:**
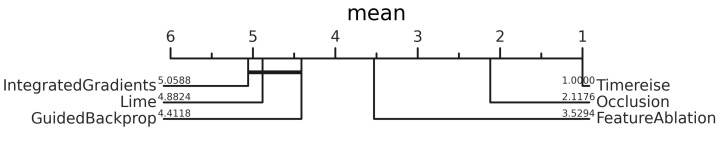
Continuity. Critical difference diagram showing the average rank of each attribution method across all datasets. The ranking is based on the average Continuity. PErturbation-based approaches show superior performance.The horizontal bars show the groups that can be concluded based on the rank of the methods. They clearly highlight the low performing set.

Figure 9 shows the corresponding critical difference diagram. It is intuitive that the Sensitivity is defined as the change in prediction when the attribution method is applied, to a slightly perturbed input, and the Continuity and the smoothness of the attribution method are connected to each other. However, it is interesting to observe the strong correlation between those two aspects.

### 5.7. Visualization

This section presents some interpretable attribution maps. The results highlight that TimeREISE produces smoother attribution maps while preserving the similar shape compared to the other attribution methods. TimeREISE builds a good compromise between the visual appearance strongly affected by the Continuity and the noise and the correctness of the feature importance values.

**Figure 10:**
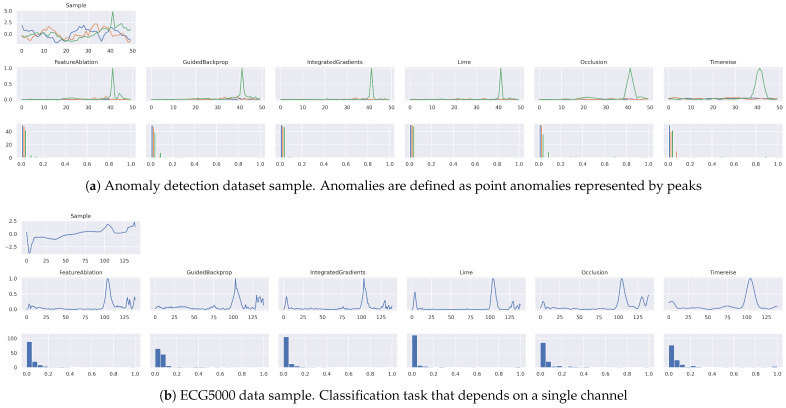
Attribution Maps. Shows the attribution maps for a single sample. The first row shows the original sample. The second row shows the actual attribution and the third row shows the histogram of the attribution scores within the given map. Generally, a large amount of low values in the histogram relates to a good separation between relevant and irrelevant features. (**a**) shows an anomalous sample in which the green peak corresponds to the anomalous signal part. Overall the attributions look similar except that TimeREISE is smoother compared to the other methods.

In Figure 10 an attribution map of every evaluated attribution map is shown. The first Figure 10a shows an anomalous sample of the Anomaly dataset. The anomaly is represented by the peak in the green signal. All methods successfully identify the peak as the most important part. However, the Occlusion and TimeREISE highlight that the neighborhood points of the peak are important. Whereas the intuition first suggests that only the peak should be highlighted, this is not correct, as changing the neighborhood points will influence the peak. Furthermore, it is visible that the attribution map provided by TimeREISE is very smooth compared to the other attributions, while preserving the relevant information.

In Figure 10b an attribution map for the ECG5000 dataset is shown. The results of all methods look similar to a certain degree. However, except for TimeREISE, the last part of the sequence is identified as features with some importance. In addition, the attribution maps include some noise. Specifically, the first negative peak in the signal is captured by the IntegratedGradients and LIME to be an important part. This is not the case for the remaining methods, and changing this part or the last part has only a minor effect on the prediction.

**Figure 11:**
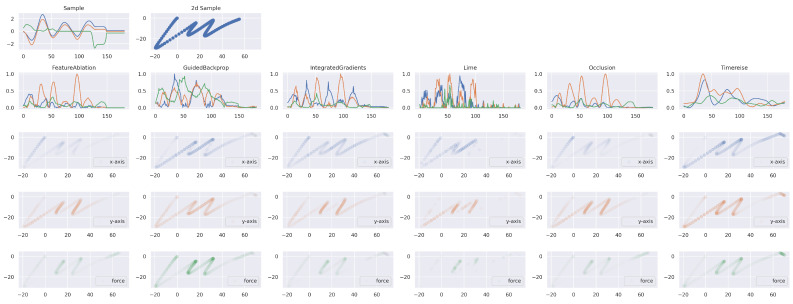
Explainable Attribution. The first row shows the time series of the character example ‘m’. The right plot corresponds to the back transformation to the original 2d space. The second row shows the attribution results for each method. The subsequent rows show the importance applied to the character for the horizontal and vertical direction as well as for the force. TimeREISE provides a smooth attribution map and assigns importance to the force channel. Besides TimeREISE, only GuidedBackprop highlights the importance of the force channel.

Figure 11 shows the results of the attribution applied to an interpretable character trajectory sample. The Figure presents the time series sample and its back transformation to 2d space. Furthermore, the attribution maps given in the second row show the smoothness of TimeREISE. One finding is that the horizontal and vertical movement are rated as more important by most methods and that the majority of important points occur within the first 100 timesteps. Interestingly, GuidedBackprop results in a surprisingly high relevance for the force. FeatureAblation and Occlusion show low importance for both the vertical movement and the pressure.

## 6. Discussion

This section discusses the results to gain a better impression of the relevance and possible applications of TimeREISE. Furthermore, the advantages and drawbacks of the existing methods are mentioned. To summarize the experiments, the datasets are grouped below based on their properties described in Table 1. Datasets with less than 1000 training samples are referenced as small datasets. In addition, a distinction is made between univariate and multivariate datasets and binary as well as multi-class datasets. A final characteristic is the sequence length, which is considered a long sequence if it exceeds 500.

During the sanity checks, TimeREISE has shown a strong dependency on the model parameters. This dependency is an indication that the method does not represent dataset characteristics, but visualizes the points considered relevant by the model. Already in the work of Mercier et al. [11], and Adebayo et al. [21] the importance of this sanity check for time series analysis and image analysis was shown. The sanity check showed strong robustness to single layer manipulation and a large drop-off in attribution map correlation when layers were manipulated sequentially. Adebayo et al. have shown that some methods provide similar attributions independent of the model parameters.

The runtime analysis also shows that TimeREISE scales are better compared to other perturbation-based approaches like Occlusion. Specifically, the long-sequence and multivariate datasets result in a dramatic increase in the runtime for perturbation-based methods and LIME. It has to be mentioned that concerning the runtime the gradient-based methods are always superior to others, as they only require a backward pass. However, TimeREISE shows superior behavior compared to the remaining methods, specifically when long sequences or high channel numbers occur. For short sequences, the runtime of TimeREISE was nearly constant. In addition, it is possible to precisely adapt the runtime of TimeREISE to the application using the number of masks to be calculated and to configure it more precisely by incorporating knowledge about the data set. One example is the detection of point anomalies, where a low density is sufficient for detection and can thus improve the runtime.

The results of the deletion and insertion test provide evidence that the attribution maps are relevant for the prediction. Especially in the deletion test, TimeREISE shows excellent results, which directly lead to performance drops when important features were removed. Besides two long sequence datasets, TimeREISE has shown superior performance across all dataset categories. The good performance in the insertion test can be explained by the fact that TimeREISE has a high Continuity, which gives contiguous time segments more uniform importance. In general, the performance of window-based approaches is good in the insertion test, since the gradients are not used to create a synthetic minimalist sample. The detection of a peak is an example of an application where the insertion test gives a false picture. Methods that use the gradients show lower importance at the timesteps adjacent to the peak, while this is not the case with TimeREISE. Intuitively, the immediately adjacent points are relevant to the existence of a peak, so they should be given some importance. Summarizing the deletion and insertion test, the results show a superior performance independent of the dataset category.

In addition, TimeREISE shows excellent results for Infidelity and Sensitivity. TimeREISE has shown the best Sensitivity values across all datasets. Concerning Infidelity, TimeREISE achieved the second-best overall performance. The best scores were achieved in long sequences. There was no specific dataset category for which the method performed worse compared to the other methods. It has to be mentioned that LIME was slightly better in the overall Infidelity, however, TimeREISE achieved more individual best scores for the datasets. Due to the mask-based design, TimeREISE has high robustness in case of insignificant changes in the input. This is also because the attribution masks created by TimeREISE have a high Continuity, which contributes to the interpretability. Despite this continuity, the attribution masks were shown to withstand the sanity check and thus offer a good compromise between explanatory power and correctness. Finally, the visualizations of the different attribution methods show that TimeREISE provides less noisy explanations. Across all datasets, TimeREISE was able to show the best Continuity values.

In summary, TimeREISE has proven to be an outstanding method for time series analysis in terms of correctness, runtime and interpretability. In addition, the customization of the hyperparameters allows easy adaptation to different aspects of use. TimeREISE can be used to determine precise attribution masks for real-time analysis with less accuracy. In addition, it is possible to customize the Continuity, and the perturbation method is interchangeable. In contrast to existing perturbation-based methods, TimeREISE can be applied without knowing the size of the relevant pattern within the data, making it more effective in cases where the solution to the question of which parts are relevant is not known.

## 7. Conclusions

This work shows that the novel attribution method TimeREISE can achieve excellent performance concerning most of the evaluated metrics across all selected datasets. The method outperforms other state-of-the-art attribution methods concerning the Continuity, Sensitivity, and causal metrics. Specifically, the deletion scores when important data are occluded show that the approach provides superb performance. Furthermore, the paper has demonstrated that the method produces smooth attribution maps that require less effort to interpret. Concerning Infidelity, our approach is on par with the state-of-the-art methods. Further, the theoretical runtime evaluation shows that the method has a better scaling compared to methods that directly depend on the number of features and is applicable to any classifier. This is further validated based on the additional runtime experiments that highlight that the increase in runtime is related to the time spent by the network to execute a forward pass. Another positive aspect is that the method does not depend on noisy gradients or internal classifier variables. Ultimately, the sanity checks highlight the dependency on the model parameters and the robustness. TimeREISE is successfully shown to strongly depend on the correctness of the network and shows significant changes when the model weights are randomized. Summarizing the metrics, TimeREISE shows superior results for Sensitivity, Continuity, deletion and insertion tests. Particularly compared to gradient-based methods, TimeREISE shows a large improvement concerning the mentioned metrics. Only insignificant changes were observed for Infidelity. Concerning the runtime the method shows superior results compared to the perturbation-based methods when the dataset covers long sequences or multiple channels. For short univariate sequences, the runtime is nearly constant and only slightly above the runtime of the other perturbation-based methods. Compared to the gradient-based methods, the runtime of TimeREISE and any other perturbation-based method is inferior.

## Figures and Tables

**Table 1:** Datasets related to critical infrastructures. Different characteristics such as the datasetsize, length, feature number and classes are covered by this selection.

Domain & Dataset	Train	Test	Steps	Channels	Classes
**Critical Manufacturing**					
Anomaly (synthetic data)	50,000	10,000	50	3	2
ElectricDevices	8926	7711	96	1	7
FordA	3601	1320	500	1	2
**Food and Agriculture**					
Crop	7200	16,800	46	1	24
Strawberry	613	370	235	1	2
**Public Health**					
ECG5000	500	4500	140	1	5
FaceDetection	5890	3524	62	144	2
MedicalImages	381	760	99	1	10
NonInvasiveFetalECG	1800	1965	750	1	42
PhalangesOutlinesCorrect	1800	858	80	1	2
**Communications**					
CharacterTrajectories	1422	1436	182	3	20
HandOutlines	1000	370	2709	1	2
UWaveGestureLibraryAll	896	3582	945	1	8
Wafer	1000	6164	152	1	2
**Transportation Systems**					
AsphaltPavementType	1055	1056	1543	1	3
AsphaltRegularity	751	751	4201	1	2
MelbournePedestrian	1194	2439	24	1	10

**Table 2:** Performance of IncpetionTime. Concerning the accuracy, and f1 scores the subsampled dataset achieves similar performance and can be used as a set of representative samples for further experiments. The results show that the subset accuracy is equal to the complete test set accuracy.

Dataset	Test Data			100 Samples		
	f1-Macro	f1-Micro	Acc	f1-Macro	f1-Micro	Acc
Anomaly	0.9769	0.9871	0.9872	0.9699	0.9797	0.9800
AsphaltPavementType	0.9169	0.9244	0.9242	0.8905	0.8991	0.9000
AsphaltRegularity	1.0000	1.0000	1.0000	1.0000	1.0000	1.0000
CharacterTrajectories	0.9940	0.9944	0.9944	1.0000	1.0000	1.0000
Crop	0.7189	0.7189	0.7281	0.7058	0.7228	0.7400
ECG5000	0.5611	0.9352	0.9436	0.6045	0.9412	0.9500
ElectricDevices	0.6286	0.6935	0.7056	0.6709	0.7602	0.7900
FaceDetection	0.6634	0.6634	0.6637	0.6779	0.6790	0.6800
FordA	0.9492	0.9492	0.9492	0.9294	0.9299	0.9300
HandOutlines	0.9464	0.9510	0.9514	0.9399	0.9493	0.9500
MedicalImages	0.7227	0.7461	0.7474	0.7086	0.7479	0.7500
MelbournePedestrian	0.9422	0.9424	0.9422	0.9635	0.9595	0.9600
NonInvasiveFetalECG	0.9400	0.9430	0.9425	0.8424	0.9240	0.9200
PhalangesOutlinesCorrect	0.8142	0.8254	0.8275	0.8849	0.8898	0.8900
Strawberry	0.9554	0.9593	0.9595	0.9672	0.9699	0.9700
UWaveGestureLibraryAll	0.9165	0.9167	0.9174	0.8525	0.8696	0.8700
Wafer	0.9954	0.9982	0.9982	1.0000	1.0000	1.0000
Average	0.8613	0.8911	0.8931	0.8593	0.8954	0.8988

**Table 3:** Deletion & Insertion. Sequential deletion of the most important points from the original input signal. Respectively, sequential insertion of the most important points starts with a sample consisting of mean values. Lower AUC scores are better for deletion. Higher AUC scores are better for insertion. AUC was calculated using classification accuracy. TimeREISE outperforms any other methods concerning the deletion and achieves the best average for both deletion and insertion. Best results are highlighted in bold.

Dataset	FeatureAblation [16]		GuidedBackprop [14]		IntegratedGrad. [15]		Lime [18]		Occlusion [17]		TimeREISE (Ours)	
	del	ins	del	ins	del	ins	del	ins	del	ins	del	ins
Anomaly	0.7731	0.9737	0.7791	0.9597	0.7786	0.9624	0.7783	0.9473	0.7714	0.9739	**0.7631**	**0.9867**
AsphaltPavementType	0.4073	0.8819	0.3930	**0.8944**	**0.3940**	0.8935	0.4622	0.8623	0.4171	0.8726	0.4135	0.8641
AsphaltRegularity	0.5857	0.9954	**0.5785**	**0.9960**	0.5817	0.9964	0.6843	0.9871	0.5901	0.9929	0.5927	0.9833
CharacterTrajectories	0.0856	0.8563	**0.0807**	0.8701	0.1091	0.8580	0.0785	0.8543	0.0878	0.8609	0.0401	**0.8809**
Crop	0.0998	0.3780	0.1402	0.3026	0.1404	0.2652	0.1096	0.3198	0.1583	0.3170	**0.0628**	**0.5065**
ECG5000	0.2104	0.8771	0.1876	0.8782	0.1208	0.8792	0.1294	0.8846	0.1176	0.8796	**0.1015**	**0.9060**
ElectricDevices	0.3086	0.5393	0.3616	0.5718	0.3178	0.5244	0.3338	0.4971	0.3524	0.5914	**0.2726**	**0.6957**
FaceDetection	0.5165	0.6760	0.2462	0.8065	0.5116	0.6660	0.6019	0.6308	0.5281	0.6691	**0.0080**	**0.9968**
FordA	0.4729	0.7816	0.4829	0.8207	0.4793	0.6834	0.4803	0.6731	0.4751	0.8493	**0.3859**	**0.9436**
HandOutlines	0.3125	0.3630	0.3137	0.3289	0.3127	0.3432	0.3153	0.3201	**0.3107**	**0.3911**	0.3485	0.3607
MedicalImages	0.1840	0.5884	0.1588	0.5645	0.1953	0.4518	0.1736	0.5622	0.1569	0.5883	**0.1229**	**0.7125**
MelbournePedestrian	0.1579	0.5967	0.2071	0.5579	0.2733	0.4579	0.1767	0.6013	0.2363	0.4763	**0.0979**	**0.6538**
NonInvasiveFetalECG	0.0424	0.1488	0.0454	0.0654	0.0405	0.0868	0.0462	0.0816	**0.0422**	0.2503	0.0894	**0.4333**
PhalangesOutlinesCorrect	0.4033	0.5072	0.4058	0.4347	0.4056	0.4437	0.4038	0.4288	0.4034	0.5616	**0.2919**	**0.6171**
Strawberry	0.5827	0.7179	0.6141	0.7100	0.6428	0.7179	0.6397	0.7087	0.5958	0.7761	**0.3882**	**0.7909**
UWaveGestureLibraryAll	0.1840	0.4243	0.1353	0.5260	0.1285	0.1452	0.1226	0.1782	0.1743	0.4669	**0.0973**	**0.5379**
Wafer	0.2740	0.7684	0.3441	0.8574	0.2603	0.8061	0.2324	0.8613	0.2642	0.7932	**0.2002**	**0.8976**
Average	0.3295	0.6514	0.3220	0.6556	0.3348	0.5989	0.3393	0.6117	0.3342	0.6653	**0.2516**	**0.7510**

**Table 4:** Infidelity. Lower values correspond to better performance. The Method names are shortened by taking only the initial character (FeatureAblation, Guided-Backpropagation, IntegratedGradients, LIME, Occlusion, TimeREISE). There are only insignificant differences between the methods. Lime shows the best performance. TimeREISE takes the second place. Best results are highlighted in bold.

Dataset	F [16]	G [14]	I [15]	L [18]	O [17]	T (Ours)
Anomaly	0.0233	0.0193	**0.0158**	0.0184	0.0222	0.0230
AsphaltPavementType	0.2126	0.2126	0.2126	0.2127	0.2126	**0.2124**
AsphaltRegularity	**0.0045**	0.0046	0.0046	0.0046	**0.0045**	**0.0045**
CharacterTrajectories	0.1399	0.1397	0.1399	0.1399	0.1399	**0.1396**
Crop	0.2967	0.3081	0.3055	**0.2966**	0.3143	0.3032
ECG5000	0.0273	0.0272	0.0257	**0.0210**	0.0236	0.0242
ElectricDevices	18.0869	**18.1047**	18.1130	18.1042	18.0854	18.1070
FaceDetection	0.0002	0.0002	0.0002	0.0002	0.0002	0.0002
FordA	0.0118	0.0118	0.0116	0.0116	0.0118	0.0118
HandOutlines	**1.6914**	1.7015	1.6932	1.6928	1.6938	1.6920
MedicalImages	0.2492	0.2492	**0.2472**	0.2486	0.2490	0.2482
MelbournePedestrian	1.2324	1.2833	1.3745	1.1959	1.3319	**1.2301**
NonInvasiveFetalECG	51.7361	51.7288	51.7252	51.7228	51.7413	**51.7072**
PhalangesOutlinesCorrect	0.4394	**0.4285**	0.4360	0.4413	0.4403	0.4405
Strawberry	0.4865	**0.4783**	0.4863	0.4849	0.4811	0.4851
UWaveGestureLibraryAll	4.9995	4.9983	4.9922	4.9996	4.9992	**4.9968**
Wafer	0.0355	0.0356	0.0355	0.0356	0.0355	**0.0352**
Average	4.6867	4.6901	4.6952	**4.6842**	4.6933	4.6859

**Table 5:** Sensitivity. Lower values correspond to better performance. The method names are shortened by taking only the initial character (FeatureAblation, Guided-Backpropagation, IntegratedGradients, LIME, Occlusion, TimeREISE). Perturbation-based approaches show superior performance. TimeREISE shows the best performance across most of the datasets. Best results are highlighted in bold.

Dataset	F [16]	G [14]	I [15]	L [18]	O [17]	T (Ours)
Anomaly	0.0574	0.0747	0.1470	0.2591	0.0664	**0.0522**
AsphaltPavementType	0.0292	0.2864	0.0358	0.4259	**0.0274**	0.0705
AsphaltRegularity	0.0288	0.2797	0.0567	0.3664	0.0274	**0.0028**
CharacterTrajectories	0.0199	0.0547	0.0705	0.1353	0.0174	**0.0076**
Crop	0.0808	0.1060	0.1702	0.1786	0.1307	**0.0411**
ECG5000	0.0301	0.0772	0.1218	0.1811	0.0248	**0.0111**
ElectricDevices	0.2069	0.2608	0.6129	0.2622	0.1949	**0.1696**
FaceDetection	0.0180	0.0204	0.0136	0.4722	0.0144	**0.0048**
FordA	0.0231	0.0384	0.0708	0.1690	0.0155	**0.0147**
HandOutlines	0.0952	0.1545	0.1203	0.1249	**0.0743**	0.1175
MedicalImages	0.0428	0.0680	0.1483	0.1754	**0.0395**	0.0406
MelbournePedestrian	0.1667	0.1363	0.1684	0.2514	0.2176	**0.0472**
NonInvasiveFetalECG	0.1142	0.1043	0.1543	0.1564	**0.0869**	0.1570
PhalangesOutlinesCorrect	0.0415	0.1442	0.1562	0.1212	**0.0390**	0.0574
Strawberry	0.0486	0.0966	**0.0506**	0.1267	0.0515	0.0698
UWaveGestureLibraryAll	0.0569	0.0535	0.2341	0.1778	**0.0373**	0.0381
Wafer	0.0252	0.0368	0.1299	0.1250	0.0141	**0.0051**
Average	0.0638	0.1172	0.1448	0.2182	0.0635	**0.0533**

**Table 6:** Continuity. Lower values correspond to better performance. The method names are shortened by taking only the initial character (FeatureAblation, Guided-Backpropagation, IntegratedGradients, LIME, Occlusion, TimeREISE). TimeREISE shows a superior performance across all datasets. Best results are highlighted in bold.

Dataset	F [16]	G [14]	I [15]	L [18]	O [17]	T (Ours)
Anomaly	0.1163	0.1444	0.1309	0.1390	0.0908	**0.0473**
AsphaltPavementType	0.0792	0.0977	0.0770	0.0765	0.0450	**0.0015**
AsphaltRegularity	0.0582	0.0703	0.0485	0.0525	0.0334	**0.0008**
CharacterTrajectories	0.0264	0.0324	0.0368	0.0619	0.0243	**0.0134**
Crop	0.1282	0.1655	0.1952	0.1741	0.0985	**0.0618**
ECG5000	0.0682	0.1000	0.1004	0.0844	0.0505	**0.0296**
ElectricDevices	0.2016	0.1840	0.1984	0.1950	0.0884	**0.0350**
FaceDetection	0.0690	0.0745	0.0613	0.0331	0.0373	**0.0161**
FordA	0.0770	0.0819	0.0959	0.1530	0.0576	**0.0083**
HandOutlines	0.0123	0.0183	0.0258	0.1501	0.0106	**0.0015**
MedicalImages	0.0923	0.1043	0.1259	0.1076	0.0602	**0.0371**
MelbournePedestrian	0.1804	0.1844	0.2217	0.1881	0.1264	**0.1052**
NonInvasiveFetalECG	0.0224	0.0650	0.0753	0.1603	0.0197	**0.0043**
PhalangesOutlinesCorrect	0.1066	0.1187	0.1525	0.1416	0.0715	**0.0496**
Strawberry	0.0720	0.0679	0.0785	0.1447	0.0676	**0.0159**
UWaveGestureLibraryAll	0.0216	0.0557	0.0816	0.1629	0.0226	**0.0038**
Wafer	0.0924	0.0957	0.1418	0.1222	0.0557	**0.0232**
Average	0.0838	0.0977	0.1087	0.1263	0.0565	**0.0267**

## Data Availability

Not applicable.
